# Orthodontic Treatment Need and Short-Term Oral Hygiene Assessment in Children Undergoing Different Early Orthodontic Treatments

**DOI:** 10.3390/children13060823

**Published:** 2026-06-17

**Authors:** Neslihan Atmaca, Murat Tozlu, Sertaç Peker, Betül Kargül

**Affiliations:** 1 Department of Pediatric Dentistry, Faculty of Dentistry, Marmara University, 34854 Istanbul, Türkiye; 2 Department of Orthodontics, Faculty of Dentistry, Marmara University, 34854 Istanbul, Türkiye; 3 Queen Mary University of London, London E1 4NS, UK

**Keywords:** mixed dentition, early orthodontic treatment, clear aligners, removable orthodontic appliances, oral hygiene status, Index of Orthodontic Treatment Need, Simplified Oral Hygiene Index

## Abstract

**Highlights:**

**What are the main findings?**

- Higher orthodontic treatment need was associated with poorer oral hygiene status during early orthodontic treatment.
- Clear aligner therapy was associated with more favorable short-term oral hygiene outcomes than removable appliances.

**What are the implications of the main findings?**

- Malocclusion severity may influence oral hygiene status during early orthodontic treatment.
- Orthodontic appliance design should be considered when planning oral hygiene management during early orthodontic treatment.

**Abstract:**

**Objectives**: Oral hygiene maintenance may be influenced by both malocclusion severity and orthodontic appliance type during early orthodontic treatment. Therefore, this study aimed to evaluate the relationship between orthodontic treatment need assessed using the Index of Orthodontic Treatment Need (IOTN) and oral hygiene status measured using the Simplified Oral Hygiene Index (OHI-S) in children undergoing early orthodontic treatment and to compare short-term oral hygiene outcomes between clear aligner and removable appliance therapies. **Methods:** Twenty-four children aged 6–12 years with anterior dental crossbite were included in this prospective observational cohort study. Patients treated with clear aligners (*n* = 12) or removable appliances (*n* = 12) were evaluated. Orthodontic treatment need was assessed using the IOTN Dental Health Component (DHC) and Aesthetic Component (AC), and oral hygiene status was evaluated using the OHI-S at baseline and day 14. Statistical analyses were performed with *p* < 0.05 considered significant. **Results:** A statistically significant positive correlation was observed between IOTN-DHC and IOTN-AC scores (rs = 0.648; *p* = 0.001). Participants with higher orthodontic treatment need had poorer oral hygiene status at baseline, and this difference remained evident at day 14. Day-14 OHI-S scores were significantly higher in the removable appliance group than in the clear aligner group (*p* = 0.039). In addition, the reduction in OHI-S scores was significantly greater in the clear aligner group (*p* = 0.043). When all participants were analyzed together, oral hygiene status improved significantly from baseline to day 14 (*p* < 0.001). **Conclusions:** Higher orthodontic treatment need was associated with poorer oral hygiene status during early orthodontic treatment. Clear aligners were associated with more favorable short-term oral hygiene outcomes than removable appliances.

## 1. Introduction

Malocclusion is one of the most common oral health problems during childhood and adolescence and may negatively affect oral function, dentofacial aesthetics, and oral health status. In addition to its functional and psychosocial consequences, malocclusion may complicate oral hygiene maintenance by promoting plaque accumulation and impairing effective cleaning of tooth surfaces [1,2]. Orthodontic treatment aims to improve dental alignment and occlusal relationships, thereby contributing to oral health, functional efficiency, and quality of life [3,4]. Accordingly, the association between malocclusion severity and oral hygiene status has attracted increasing attention in orthodontic research [5].

The Index of Orthodontic Treatment Need (IOTN) is one of the most widely used indices for the standardized assessment of malocclusion severity and orthodontic treatment need [6,7]. The index consists of two complementary components: the Dental Health Component (IOTN-DHC), which evaluates the clinical significance of malocclusion according to occlusal traits, and the Aesthetic Component (IOTN-AC), which assesses the aesthetic impact of malocclusion using a standardized photographic scale. Together, these components provide a comprehensive evaluation of orthodontic treatment need from both functional and aesthetic perspectives [8,9].

Early orthodontic treatment performed during the mixed dentition period aims to intercept developing malocclusions and improve occlusal relationships at an early stage. Assessing orthodontic treatment need during this period is clinically important, as malocclusions identified in mixed dentition may influence both treatment planning and oral hygiene management. Traditionally, removable appliances have been widely used during early orthodontic treatment; however, clear aligners have recently gained increasing popularity as an alternative treatment modality during the mixed dentition period [10]. Appliance-related factors such as acrylic coverage, retentive components, surface characteristics, and patient compliance may influence plaque accumulation and oral hygiene maintenance throughout treatment [11,12].

Although previous studies have investigated the associations between malocclusion severity, orthodontic treatment need, and oral health outcomes, evidence specifically addressing oral hygiene status during interceptive orthodontic treatment in mixed-dentition children is still relatively limited and context-dependent [13,14,15]. In addition, comparative data regarding short-term oral hygiene changes associated with clear aligners and removable appliances during early orthodontic treatment remain scarce. Therefore, the present study focused on a specific clinical setting by evaluating children undergoing interceptive orthodontic treatment for anterior dental crossbite during the mixed dentition period. To the best of our knowledge, this is one of the few studies to directly compare short-term oral hygiene outcomes between clear aligners and removable appliances in mixed-dentition children undergoing interceptive orthodontic treatment.

The present study aimed to evaluate the relationship between orthodontic treatment need assessed using the IOTN and oral hygiene status measured using the OHI-S in children undergoing early orthodontic treatment. By focusing on mixed-dentition children treated for anterior dental crossbite and comparing short-term oral hygiene outcomes between clear aligners and removable appliances, this study addresses an underexplored area in the literature. In addition, oral hygiene changes associated with clear aligners and removable appliances were compared during the treatment period. The null hypothesis was that neither orthodontic treatment need nor appliance type would significantly influence short-term oral hygiene status.

## 2. Materials and Methods

### 2.1. Ethical Approval

This study was conducted in accordance with the ethical principles of the Declaration of Helsinki. Ethical approval was obtained from the Clinical Research Ethics Committee of Marmara University Faculty of Medicine (Protocol Code: 09.2023.1447; approval date: 3 November 2023). The study was also registered at ClinicalTrials.gov (NCT07331090) (9 January 2026). Prior to participation, parents or legal guardians were informed about the aims and procedures of the study, and written informed consent was obtained for all participants.

### 2.2. Study Design and Setting

This prospective observational cohort study was conducted between March 2024 and May 2025 at a university-based dental clinic. Children aged 6–12 years who were scheduled to undergo early orthodontic treatment with either clear aligners or removable appliances for anterior dental crossbite were included in the study. Treatment modality was determined according to routine clinical practice rather than randomization. Treatment selection was based on the treating orthodontist’s clinical judgment, appliance suitability, and patient/family preference. Therefore, allocation was not random and may have introduced selection bias.

The study was reported in accordance with the STROBE (Strengthening the Reporting of Observational Studies in Epidemiology) statement [16]. The 14-day follow-up period was selected to evaluate short-term oral hygiene changes during the initial adaptation phase following appliance placement, as previous evidence suggests that the most pronounced treatment-related changes and patient adaptation occur during the first days and weeks of orthodontic treatment [17]. All participants were followed prospectively, and outcome measures were assessed before treatment initiation and on the 14th day of treatment.

### 2.3. Sample Size

A priori sample size calculation was performed using G*Power software (version 3.1.9.7; Heinrich Heine University, Düsseldorf, Germany). With an alpha level of 0.05, a statistical power of 80%, and an effect size (f) of 0.25, the analysis indicated that a minimum of 24 participants would be required [18]. Since a specific pilot study or an identical previous trial investigating the exact same primary outcome configuration was not available in the literature to provide a direct baseline effect size, we adopted a standardized and conservative methodological approach. According to Cohen’s conventions for ANOVA-based analyses, an effect size of f = 0.25 represents a medium effect size and is commonly used in the absence of empirical estimates from previous studies. Therefore, this value was adopted for the a priori sample size calculation to ensure adequate statistical power while avoiding overestimation of the expected treatment effect.

### 2.4. Participants

**Figure 1:**
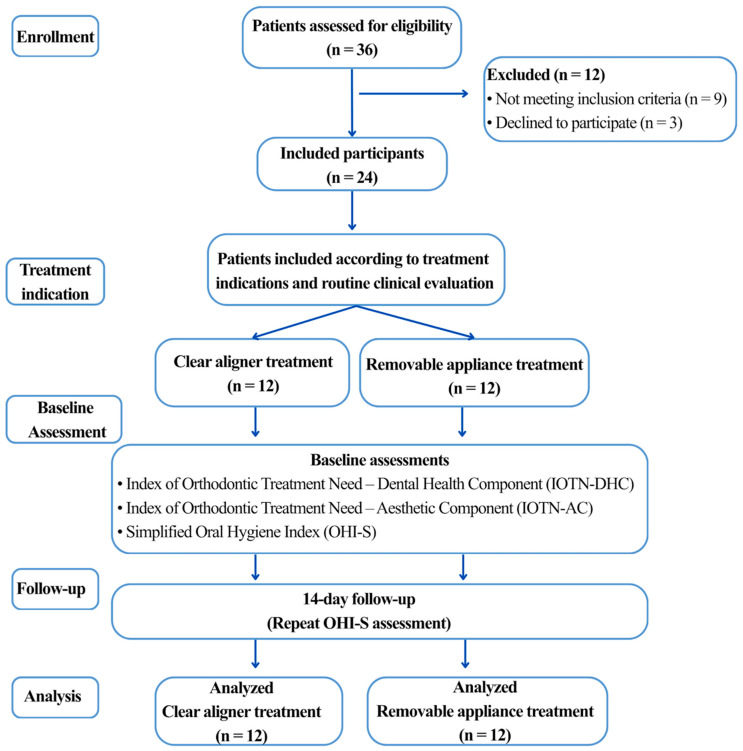
Flow diagram of participant recruitment, treatment indication, follow-up, and analysis procedures. All participants completed the 14-day follow-up and were included in the final analysis.

Twenty-four children who met the inclusion criteria were included in the study. Based on routine clinical evaluation and treatment indications, patients indicated for clear aligner treatment (*n* = 12) or removable appliance treatment (*n* = 12) were included in the study. All participants completed the study protocol and follow-up evaluations. Inclusion criteria were as follows: children aged 6–12 years in the mixed dentition period requiring early orthodontic treatment for dental anterior crossbite and having complete baseline and follow-up assessments. Exclusion criteria included skeletal malocclusion, craniofacial anomalies or syndromes, previous orthodontic treatment, systemic diseases affecting oral health, use of medications that could influence oral health conditions, and active gingival or periodontal disease. Participant recruitment, treatment indication, follow-up, and analysis procedures are summarized in Figure 1.

### 2.5. Orthodontic Treatment Need Assessment

Orthodontic treatment need was evaluated using the Index of Orthodontic Treatment Need (IOTN), which consists of the Dental Health Component (DHC) and the Aesthetic Component (AC). The IOTN-DHC was used to assess occlusal characteristics and orthodontic treatment need, whereas the IOTN-AC evaluated the aesthetic appearance of the anterior dentition using a standardized photographic scale [6].

For descriptive and comparative analyses, IOTN-DHC grades 1–2 were considered little treatment need, grade 3 as borderline treatment need, and grades 4–5 as high treatment need according to the severity of malocclusion. IOTN-AC scores were categorized as follows: scores of 1–4 indicated little or no treatment need, scores of 5–7 represented borderline treatment need, and scores of 8–10 indicated high treatment need [6,19,20].

All orthodontic assessments were performed under standardized clinical conditions by a single calibrated researcher. To assess intra-examiner reliability, all participants were re-evaluated two weeks after the initial assessment. Intra-examiner reliability demonstrated excellent agreement (ICC = 0.97).

### 2.6. Oral Hygiene Assessment

Oral hygiene status was evaluated using the Simplified Oral Hygiene Index (OHI-S), which combines the Simplified Debris Index (DI-S) and the Simplified Calculus Index (CI-S) [21]. Clinical examinations were performed under standardized conditions using a dental mirror and probe by a single calibrated researcher. The examiner reviewed the OHI-S scoring criteria before study initiation to ensure consistent application of the index throughout the study.

The OHI-S assessment was conducted according to the criteria described by Greene and Vermillion on selected anterior and posterior tooth surfaces. In mixed dentition, the buccal surfaces of the maxillary molars and central incisor and the lingual surfaces of the mandibular molars and central incisor were examined. Debris and calculus accumulation on each surface were scored from 0 to 3 according to the amount of deposits present. A score of 0 indicated the absence of debris or calculus, whereas scores of 1, 2, and 3 represented increasing amounts of debris and calculus accumulation on the tooth surface [21,22].

The DI-S and CI-S scores obtained from the examined tooth surfaces were averaged separately, and the total OHI-S score was calculated as the sum of these values. OHI-S measurements were recorded at baseline and on the 14th day of treatment. A 14-day follow-up period was chosen to capture early behavioral adaptation to orthodontic appliances, as this period is considered sufficient to detect initial changes in oral hygiene status and plaque accumulation, and has been frequently used in short-term orthodontic studies evaluating oral hygiene outcomes.

### 2.7. Orthodontic Treatment Procedures

Participants were treated with either clear aligners or removable orthodontic appliances according to the treatment plan established by the orthodontist. Before treatment initiation, standardized oral hygiene instructions were provided to all participants.

Children in the clear aligner group were treated using the Maraligner Clear Dental Aligner system (Marmara Digital Technology, Istanbul, Türkiye). Digital intraoral scans were obtained using a TRIOS 3 intraoral scanner (3Shape A/S, Copenhagen, Denmark), and individualized treatment plans were prepared for each participant. Composite attachments were bonded using a transfer template when required to improve retention and facilitate planned tooth movement. Participants were instructed to wear the aligners for approximately 20 h per day.

Children in the removable appliance group were treated using spring-activated removable appliances fabricated on acrylic plates adapted to the palatal mucosa. All appliances were manufactured in the same laboratory according to a standardized design. The appliance design incorporated Adams clasps on the maxillary molars, drop clasps between premolar or primary molar teeth, and a U-shaped vestibular arch. Retentive wire components were fabricated from 0.7-mm stainless steel round wire, and a 0.5-mm stainless steel spring positioned palatally was used for anterior crossbite correction. Posterior acrylic bite blocks were used to eliminate occlusal interference during treatment. Participants were instructed to wear the removable appliances for approximately 20 h daily.

### 2.8. Statistical Analysis

Descriptive statistics were calculated for all variables. Continuous data were presented as mean ± standard deviation (SD) and median with interquartile range (IQR), while categorical variables were expressed as frequencies and percentages. The normality of data distribution was assessed using the Shapiro–Wilk test. According to the distribution characteristics, appropriate parametric or non-parametric methods were selected for further analysis.

For comparisons of clinical parameters between the Clear Aligner and Removable Appliance groups, the Independent Samples *t*-test was used for normally distributed data, whereas the Mann–Whitney U test was used for non-normally distributed data. Categorical variables, such as gender and IOTN treatment need categories, were compared using Fisher’s Exact test. The Wilcoxon Signed-Rank test was used to evaluate changes in OHI-S scores between baseline and the 14th day across the entire study population.

The relationship between the IOTN Dental Health Component (DHC) and Aesthetic Component (AC) was evaluated using Spearman’s rank correlation test. Furthermore, the Mann–Whitney U test was used to compare OHI-S scores across different IOTN treatment need categories. Statistical significance was set at *p* < 0.05 for all analyses. All statistical procedures were performed using IBM SPSS Statistics software (Version 21.0).

## 3. Results

**Figure 2:**
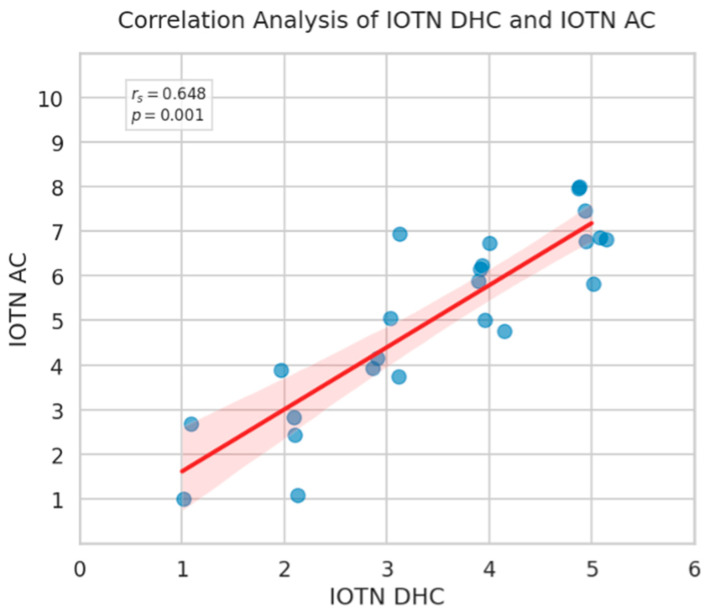
Scatter plot of the correlation between IOTN-DHC and IOTN-AC scores (*n* = 24). Spearman’s rank correlation test was used for non-parametric data, and *p* < 0.05 was considered statistically significant.

The distribution of the Index of Orthodontic Treatment Need (IOTN) Dental Health Component (DHC) and Aesthetic Component (AC) values for the 24 participants is presented in Table 1. The mean DHC score was 3.7 ± 0.8 (Median: 4.0), and the mean AC score was 6.4 ± 1.8 (Median: 6.5). Spearman’s rank correlation test revealed a statistically significant, strong positive correlation between IOTN-DHC and IOTN-AC scores (r_s_ = 0.648; *p* = 0.001). The correlation between DHC and AC scores is presented in Figure 2.

There were no statistically significant differences between the Clear Aligner (*n* = 12) and Removable Appliance (*n* = 12) groups regarding sex, age, baseline IOTN-DHC, or IOTN-AC scores (*p* > 0.05). Baseline OHI-S scores were similar between groups (*p* = 0.792). However, day-14 OHI-S scores were significantly higher in the Removable Appliance group (0.68 ± 0.57) compared to the Clear Aligner group (0.22 ± 0.25) (*p* = 0.039; r = 0.42). Furthermore, the reduction in OHI-S scores was significantly greater in the Clear Aligner group (*p* = 0.043; Cohen’s d = 0.85) (Table 2).

**Figure 3:**
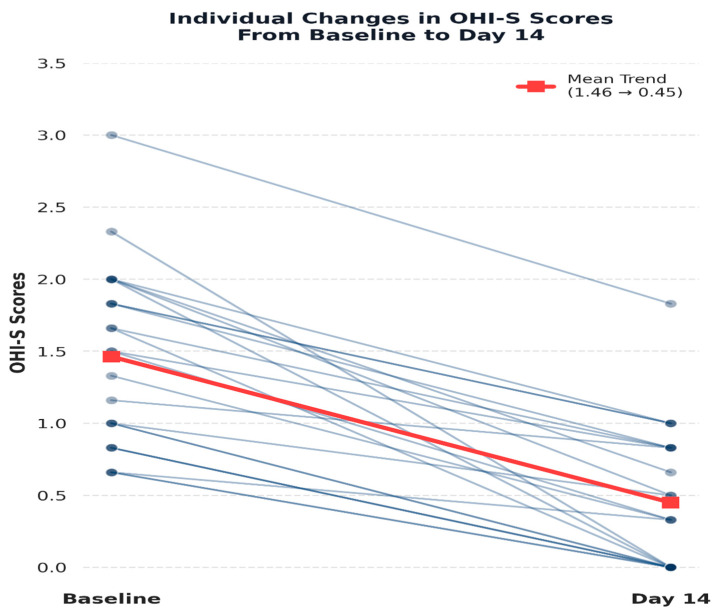
Individual trajectories of OHI-S score changes from baseline to Day 14 (*n* = 24). The thin blue lines represent the longitudinal changes for each individual participant, illustrating the variance, data distribution, and clinical response patterns within the sample. The thick red line with square markers denotes the overall mean trend, highlighting a substantial and consistent reduction in OHI-S scores (1.48 ± 0.60 to 0.41 ± 0.50; *p* < 0.001) following the intervention.

When all participants were evaluated regardless of treatment group (*n* = 24), a statistically significant decrease was observed between baseline OHI-S scores [Median: 1.54 (IQR: 0.96–1.92)] and day-14 [Median: 0.38 (IQR: 0.00–0.83)] OHI-S scores (*p* < 0.001; r = 0.84). The distribution of individual OHI-S score changes from baseline to Day 14 is illustrated in Figure 3.

When the DHCs and ACs were evaluated independently, significant differences in oral hygiene parameters were observed across treatment need categories (Table 3). Regarding the IOTN-DHC, participants categorized as having a high treatment need (*n* = 16) exhibited significantly higher baseline OHI-S scores (Median: 1.83) and day-14 OHI-S scores (Median: 0.75) compared to those in the little/borderline treatment need group (*n* = 8), with *p*-values of 0.001 (r = 0.67) and 0.003 (r = 0.61), respectively.

Similarly, for the IOTN-AC, individuals with a high treatment need (*n* = 8) showed significantly higher baseline OHI-S scores (Median: 1.92) compared to the little/borderline group (*n* = 16, Median: 1.08; *p* = 0.013; r = 0.51). In addition, participants with high aesthetic treatment needs demonstrated significantly greater improvement in OHI-S scores compared to the little/borderline treatment need group (Median: −1.17 vs. −0.83, respectively; *p* = 0.027; r = 0.45) (Table 3).

## 4. Discussion

Early orthodontic treatment during the mixed dentition period plays an important role in the management of developing malocclusions and the maintenance of oral health [10]. Although previous studies have evaluated the relationship between malocclusion severity and oral health parameters, evidence regarding oral hygiene changes according to orthodontic treatment need and appliance type during early orthodontic treatment remains limited, particularly in children treated during the mixed dentition period. Therefore, the present study investigated the relationship between orthodontic treatment need assessed using the IOTN and oral hygiene status assessed using the OHI-S in children undergoing early orthodontic treatment with clear aligners and removable appliances.

The findings of the present study demonstrated that children with greater orthodontic treatment need, particularly according to the IOTN-DHC, exhibited poorer oral hygiene status at baseline, and this difference remained evident at day 14. However, the magnitude of change in OHI-S scores during the observation period did not differ significantly between IOTN-DHC categories. Therefore, the present findings primarily support an association between orthodontic treatment need and oral hygiene status rather than an influence on short-term oral hygiene changes occurring during treatment. In addition, children treated with clear aligners were associated with more favorable short-term OHI-S outcomes than those treated with removable appliances. Furthermore, oral hygiene status improved significantly during the observation period in the overall study population. Accordingly, the null hypothesis was rejected.

Consistent with the findings of the present study, Salim et al. reported that children with higher orthodontic treatment need, particularly those with IOTN grades 3–5, demonstrated poorer oral hygiene status as assessed using the OHI-S [14]. Similarly, the present findings demonstrated that children with greater orthodontic treatment need, especially according to the IOTN-DHC, exhibited poorer oral hygiene status at baseline, and this difference remained evident throughout the observation period. Supporting these findings, previous studies have suggested that increased malocclusion severity and specific occlusal characteristics, particularly crowding and increased overjet, may negatively affect oral hygiene maintenance and promote plaque accumulation in children and adolescents undergoing orthodontic treatment [13,15].

The relationship between increased orthodontic treatment need and poorer oral hygiene status may be explained by the greater difficulty in maintaining effective plaque control in the presence of malocclusion-related occlusal irregularities. Increased plaque-retentive areas, impaired self-cleansing mechanisms, and limited accessibility during tooth brushing may contribute to plaque accumulation and compromised oral hygiene in children with more severe malocclusions. In addition, these findings further emphasize the importance of early orthodontic assessment and preventive oral hygiene reinforcement in children with increased orthodontic treatment need [23].

The findings of the present study are consistent with previous studies reporting more favorable oral hygiene outcomes in patients treated with clear aligners compared with removable orthodontic appliances. This may be attributed to the removable design of clear aligners, which facilitates routine tooth brushing and oral hygiene procedures while reducing plaque-retentive areas during treatment. This interpretation is supported by previous reviews reporting that clear aligner therapy may facilitate plaque control and periodontal health maintenance during orthodontic treatment [24]. In contrast, removable appliances incorporating acrylic coverage and retentive wire components may complicate effective tooth cleaning and increase plaque accumulation around appliance margins [25]. In addition, improved oral hygiene outcomes in the clear aligner group may also be associated with patient-related behavioral factors, including treatment compliance and motivation; however, these variables were not quantitatively assessed in the present study.

Similarly, Chen et al. reported that both clear aligners and traditional removable appliances may influence the oral microbiome and periodontal health parameters in children during the mixed dentition period. Greater microbial and periodontal alterations were observed in patients treated with traditional removable appliances, further emphasizing the influence of appliance design on oral hygiene maintenance during orthodontic treatment [12]. These findings support the results of the present study, in which more favorable short-term oral hygiene outcomes were observed in children treated with clear aligners than in those treated with removable appliances.

Furthermore, oral hygiene status improved significantly in the overall study population, regardless of treatment modality. This improvement may be associated with standardized oral hygiene instructions provided before treatment, increased oral health awareness during the early treatment period, regular clinical follow-up, and parental supervision throughout treatment. Therefore, the observed short-term improvements should be interpreted cautiously and may not be attributable exclusively to the orthodontic appliance itself. Previous studies have similarly demonstrated that repeated reinforcement and follow-up communication may improve oral hygiene compliance during orthodontic treatment, even over relatively short observation periods [26].

The use of OHI-S as an outcome measure over a 14-day observation period warrants consideration. Given the relatively short duration of the intervention, changes in OHI-S are likely to reflect primarily variations in the debris index component (DI-S), whereas the calculus index component (CI-S) would be expected to remain largely unchanged. Accordingly, short-term improvements in oral hygiene status may be driven predominantly by reductions in plaque and soft deposits rather than changes in calculus accumulation. In addition, professional prophylaxis was not performed before baseline assessment. As a result, pre-existing calculus deposits may have contributed to baseline OHI-S scores and may have reduced the sensitivity of the index to detect short-term changes. These factors should be considered when interpreting the study findings.

Regarding the aesthetic component of the IOTN, participants with higher aesthetic treatment need demonstrated poorer baseline oral hygiene status but greater improvement in OHI-S scores throughout the observation period. This finding may suggest that visible aesthetic concerns associated with malocclusion could increase patients’ motivation toward oral hygiene practices during treatment. Similarly, previous studies based on the sociodental model have demonstrated that psychosocial impact, perceived treatment need, and behavioral propensity may influence patients’ perceptions and responses to orthodontic treatment [27]. In addition, the positive correlation identified between IOTN-DHC and IOTN-AC scores suggests that increased functional treatment need may also be associated with greater perceived aesthetic impairment in children during the mixed dentition period.

From a clinical perspective, the findings of the present study highlight the importance of individualized oral hygiene management during early orthodontic treatment, particularly in children with increased orthodontic treatment need and those treated with removable appliances. Children with greater orthodontic treatment need may require more intensive oral hygiene monitoring because malocclusion-related occlusal irregularities may complicate effective plaque control. In addition, removable appliances incorporating acrylic coverage and retentive wire components may require repeated preventive reinforcement, parental supervision, and appliance-cleaning instructions during the initial treatment period. Therefore, individualized oral hygiene support and regular professional monitoring may help minimize plaque accumulation and maintain oral health throughout treatment.

There are several limitations that should be considered when interpreting the findings of the present study. Because treatment allocation was based on routine clinical decision-making rather than randomization, the possibility of selection bias cannot be completely excluded. Treatment selection reflected clinical judgment, appliance suitability, and patient/family preference; therefore, residual confounding related to behavioral characteristics, motivation, or treatment compliance may have influenced the observed oral hygiene outcomes. Consequently, the more favorable oral hygiene findings observed in the clear aligner group should not be interpreted as being exclusively attributable to appliance-related factors. The single-center design, relatively small sample size, and short follow-up period may also limit the generalizability of the findings and restrict the evaluation of long-term outcomes. In addition, all participants presented with anterior dental crossbite and required early orthodontic treatment; therefore, the findings may not be directly generalizable to children undergoing interceptive treatment for other types of malocclusions. Furthermore, oral hygiene status was assessed using a single clinical index, while microbiological parameters associated with plaque accumulation were not evaluated. Although the OHI-S includes both debris and calculus components, its responsiveness to short-term changes should be interpreted with caution. Nevertheless, OHI-S was selected because it is a widely used and validated clinical index for assessing overall oral hygiene status in both permanent and mixed dentition populations. Furthermore, a formal intra-examiner reproducibility assessment was not performed for OHI-S measurements, which should be considered when interpreting the findings. In addition, patient-reported oral hygiene behaviors, treatment compliance, and actual appliance wear time were not quantitatively monitored throughout the observation period.

Despite these limitations, the present study has several strengths. Although previous studies have investigated the relationship between orthodontic treatment need and oral hygiene status, limited evidence is available specifically in children undergoing early orthodontic treatment during the mixed dentition period. Moreover, the prospective cohort design, baseline comparability between treatment groups, and standardized clinical assessment protocol strengthen the internal validity of the study. However, due to the non-randomized design, relatively small sample size, and short follow-up period, these findings should be interpreted as preliminary. Further multicenter studies with larger sample sizes and longer follow-up periods are warranted to confirm the present findings.

## 5. Conclusions

Clear aligner therapy was associated with more favorable short-term oral hygiene outcomes than removable appliances during the initial phase of early orthodontic treatment. In addition, increased orthodontic treatment need was associated with poorer oral hygiene status at baseline, and this difference remained evident throughout the observation period, highlighting the importance of individualized oral hygiene monitoring throughout treatment. Nevertheless, due to the non-randomized design, relatively small sample size, and short follow-up period, the present findings should be interpreted as preliminary.

## Figures and Tables

**Table 1:** Distribution of AC and DHC values (*n* = 24).

	Mean	SD	Median	IQR
**DHC**	3.7	0.8	4.0	(3.0–4.0)
**AC**	6.4	1.8	6.5	(5.5–8.0)

AC: Aesthetic component, DHC: Dental health component, SD: Standard deviation, IQR: interquartile range.

**Table 2:** Comparison of demographic and clinical parameters between treatment modalities.

	Category	Clear Aligner (*n* = 12)	Removable Appliance (*n* = 12)	*p*-Value
**Gender *n* (%)**	Male	7 (58.3%)	6 (50.0%)	>0.999 *
	Female	5 (41.7%)	6 (50.0%)	
**DHC Category** ***n*** **(%)**	Little/Borderline Treatment Need	4 (33.3%)	4 (33.3%)	>0.999 *
	High Treatment Need	8 (66.7%)	8 (66.7%)	
**AC Category** ***n*** **(%)**	Little/Borderline Treatment Need	7 (58.3%)	9 (75.0%)	0.667 *
	High Treatment Need	5 (41.7%)	3 (25.0%)	
	**Clear Aligner (*n* = 12)**		**Removable Appliance (*n* = 12)**	***p*** **-value**
	**Mean ± SD** **(Median [IQR])**		**Mean ± SD** **(Median [IQR])**	
**Age (Years)**	8.9 ± 1.2 (9.0 [8.0–9.5])		8.8 ± 1.5 (8.5 [8.0–10.0])	0.882 **
**Baseline OHI-S**	1.43 ± 0.57 (1.42 [1.0–2.0])		1.50 ± 0.68 (1.58 [0.83–1.83])	0.792 **
**Day-14 OHI-S**	0.22 ± 0.25 (0.17 [0.0–0.42])		0.68 ± 0.57 (0.83 [0.0–1.0])	**0.039** ***
**Change in OHI-S scores**	−1.21 ± 0.60 (−1.09 [−1.58–−0.83])		−0.82 ± 0.21 (−0.83 [−0.92–−0.75])	**0.043** **
**IOTN-DHC**	3.8 ± 0.7 (4.0 [3.0–4.0])		3.6 ± 0.9 (4.0 [3.0–4.0])	0.671 ***
**IOTN-AC**	6.58 ± 1.93 (6.50 [5.0–8.0])		6.25 ± 1.71 (6.50 [6.0–7.5])	0.799 ***

Note: Data are presented as *n* (%), mean ± standard deviation and median [IQR]. *p* < 0.05 was considered statistically significant. * Fisher’s Exact Test. ** Independent Samples *t*-test. *** Mann–Whitney U test.

**Table 3:** Comparison of OHI-S scores between IOTN categories (DHC and AC).

	IOTN-DHC		*p*-Value *	
	Little/Borderline Treatment Need (*n* = 8) Median (IQR)	High Treatment Need (*n* = 16) Median (IQR)		Hodges–Lehmann 95% CI
**Baseline OHI-S**	0.83 (0.75–1.00)	1.83 (1.42–2.00)	**0.001**	**−1.17–−0.34**
**Day-14** **OHI-S**	0.00 (0.00–0.00)	0.75 (0.33–0.92)	**0.003**	**−0.83–−0.33**
**Change in** **OHI-S scores**	−0.83 (−0.92–−0.66)	−1.00 (−1.26–−0.83)	0.172	−0.16–0.51
	**IOTN-AC**		***p*** -value *	
	**Little/Borderline Treatment Need (*n* = 16) Median (IQR)**	**High Treatment Need (*n* = 8) Median (IQR)**		**Hodges–Lehmann 95% CI**
**Baseline OHI-S**	1.08 (0.83–1.75)	1.92 (1.58–2.17)	**0.013**	**−1.17–−0.17**
**Day-14** **OHI-S**	0.17 (0.00–0.83)	0.58 (0.17–0.92)	0.291	−0.67–0.17
**Change in** **OHI-S scores**	−0.83 (−1.00–−0.66)	−1.17 (−1.42–−0.92)	**0.027**	**0.00–0.67**

* *p*-values were calculated using the Mann–Whitney U test. Abbreviations: IOTN-DHC, Index of Orthodontic Treatment Need–Dental Health Component; IOTN-AC, Index of Orthodontic Treatment Need–Aesthetic Component; OHI-S, Simplified Oral Hygiene Index; IQR, interquartile range.

## Data Availability

The data that support the findings of this study are not publicly available due to privacy restrictions but are available from the corresponding author upon reasonable request.
